# Care interaction adding challenges to old patients’ well-being during surgical hospital treatment

**DOI:** 10.3402/qhw.v10.28830

**Published:** 2015-10-23

**Authors:** Lisbeth Uhrenfeldt, Mette Terp Høybye

**Affiliations:** 1 Horsens Hospital Research Unit, Horsens, Denmark; 2 Department of Health, Science and Technology, Aalborg University, Aalborg, Denmark; 3 Research Unit, Elective Surgery Centre, Silkeborg Regional Hospital, Silkeborg, Denmark

**Keywords:** Well-being, qualitative, health, patient-centred care, continuity of care, ethnography, nursing care, hermeneutics

## Abstract

Today, hospitals offer surgical treatment within a short hospital admission. This brief interaction may challenge the well-being of old patients. The aim of this study was to explore how the well-being of old hospitalized patients was affected by the interaction with staff during a fast-track surgical treatment and hospital admission for colon cancer. We used an ethnographic methodology with field observations and unstructured interviews focusing on one patient at a time (*n=*9) during a full day; the hours ranging from 7:45 a.m. to 8 p.m. Participants were between 74 and 85 years of age and of both sexes. The study was reported to the Danish Data Protection Agency with reference number (2007-58-0010). The encounter between old patients and the staff was a main theme in our findings elucidating a number of care challenges. The identified care challenges illustrated “well-being as a matter of different perspectives,” “vulnerability in contrast to well-being,” and “staff mix influencing the care encounter.” The experience of well-being in old cancer patients during hospital admission was absent or challenged when staff did not acknowledge their individual vulnerability and needs.

Hospitalized patients are challenged in different ways: being away from loved ones and everyday routines (Uhrenfeldt, & Høybye, 2014) and having irrational thoughts or suppositions, psychological distress, and educational needs (Dhingra et al., 2013). In addition, patients may experience transfers between hospitals and wards, or long geographical distances that challenge them and/or their significant others (Uhrenfeldt et al., 2013). Also, patients’ experience of the actual care and treatment is influenced by how respectful, thoughtful, compassionate, holistic, and individualized the care provided is (Albarran et al., 2011).

During the last decade, hospital stays in Denmark have on average shortened from 6–10 days to 2–3 days (Kehlet & Willmore, 2005) due to a multimodal package, known as “fast-track” techniques, to decrease post-surgical organ dysfunction and complications, and to improve postoperative recovery. Enhanced recovery after fast-track surgery entails a multidisciplinary approach to perioperative care to permit earlier discharge from hospital (Kehlet, 2006). However, the overall quick process may pose particular challenges in the provision of individualized care to older surgical patients.

Major surgery induces profound physiological responses in patients; frequent sequelae include pain, nausea, ileus, increased cardiac demands, and impaired pulmonary function; which can lead to delayed mobilization, prolonged hospital stay, and postoperative complications (Kitching & O'Neill, 2009). Complications like these may require additional care interventions by hospital staff to deliver optimal and respectful care. A Swedish phenomenographic analysis of hospital nurses outlined four ways of understanding the nurse's role in the interaction with the surgical patient: (1) assisting the patient to follow instructions about medical treatment, and maintaining routines; (2) providing information, service, and coordination; (3) helping and supporting patients as vulnerable individuals; and (4) encouraging patients to participate in the caring process with individual needs and personal resources (Jangland, Larsson, & Gunningberg, 2011).

Older patients may find the hospital environment especially foreign and challenging (Shapiro, Clevenger, & Evans, 2012), as their level of physical function is most often significantly impaired by their illness. Short hospital stays produce new demands for nurses to use their competencies and discernment (Uhrenfeldt, & Hall, 2007) with patients concerned about their well-being and safety (Hamström, Kankkunen, Suominen, & Meretoja, 2012). Older patients may have particular needs to consider in the caring practices of fast-track procedures to ensure their experience of well-being. This study explores how the well-being of old hospitalized patients is affected by the interaction with staff during surgical hospital treatment for colon cancer.

## Theoretical framework

Health and well-being has been defined by the World Health Organization (1989) as physical, mental, and social well-being (Särvimäki, 2006). In our study, well-being is understood from an existential perspective where well-being is a subjective feeling or experience of feeling good and being satisfied (Särvimäki, 2006). This existential understanding is based on Heidegger's definition of *Dasein* or “to be a being and a human being” and the differences herein (Heidegger, 1978), as it aims to understand what it is to be well. Human beings are whole beings whose experience can only be understood as such (Särvimäki, 2006), which means that well-being must be studied as a complex, existential human experience.

Caring is central to the delivery of health services and has been institutionalized as such. However, *caring* is in its essence a way to be in the world and to be with others in that same world as “an existential *a priori”* (Särvimäki, 2006, p. 7). Therefore, humans need to balance life between a restful dwelling and an active mobility; in a reach for a personal situational flexible movement in a “dwelling-mobility” (Galvin & Todres, 2011, p. 1). By “dwelling,” the person experiences a positive “at homeness” in being present, experiencing comfort, peace, kinship, and belonging with present-centredness (Galvin & Todres, 2011, p. 3). A contrast to the positive dwelling is suffering. Suffering occurs when dwelling is replaced by a feeling of being “exiled,” being in alienated isolation, feeling like an object rather than a person, and with bodily discomfort and pain (Galvin & Todres, 2013, p. 99). Mobility is the opposite of dwelling and both are necessary for well-being and to be able to make a “future orientation,” where one is energized by the excitement and vitality of positive changes in valued projects (Galvin & Todres, 2011, p. 4). Dwelling mobility contains the renewal and grounded vibrancy of an interpersonal contact with layered continuity and a personal experience of “mirror-like multi-functional fullness” (Galvin & Todres, 2011, p. 6).

As was stressed by Mol, Moser, and Pols (2010), p. 14), good care is a collective achievement and involves “persistent tinkering in a world full of complex ambivalence and shifting tensions”. Such active collaboration is, as Mol (2008) has extensively argued, at the core of providing good care. Tracing the *logic of care* (Mol, 2008) through the shifting interactions between patients and staff provides a way to grasp the ambiguities of care practices.

## Design

The study was inspired by ethnographic qualitative research strategies of participant observation, particularly the “go-along” method (Kusenbach, 2003). The first author went along and followed nine patients in their daily activities during 1 day (from 7:45 a.m. to 8 pm) of their hospital stay during 2012. The researcher observed the patients’ interaction with staff, with other patients, and with relatives. The surgical ward was a 44-bed surgical ward in an urban teaching hospital in Denmark.

The ethnographic approach was chosen to explore the challenges experienced by old surgical patients in interactions with the nursing staff at the ward. The approach, which contained hours of silent observations, observations, and dialogue, as well as mutual interpretation of statements, provided an opportunity to stay with the patients over a period of time while observing the patients’ interactions with staff and thereby reaching a deeper understanding of the patients’ experience of these.

### Ethical considerations

The Central Denmark Regional Research council registered the protocol (Data Protection Agency, journal no. 2007-58-0010). The study purpose and design was presented and introduced for staff and leaders together with the prepared patient handout with a short informative text on the study aim, and precautions to ensure patient anonymity. The gatekeeper to participating patients was the head nurse who selected and contacted the patients.

### Data collection

Observation was the guiding element in the actual research sessions of participant observation (Holy, 1984) with “go-along” participation in the clinical context serving as means to be able to observe (Kusenbach, 2003). Observations were recorded in a fieldwork diary with patient number, date, time of the day, and with which staff members the patient's interactions occurred. Interviews were spontaneously developed during such observations.

Patients (*n=*10) were recruited by the head nurse who contacted patients the day before or on the morning of a research day and provided both oral and written information about the study. Patients were asked to provide written consent if they agreed to participate. When a patient consented to participate in the study, the head nurse contacted the researcher. One patient withdrew her consent after meeting the researcher, as she found herself too exhausted to meet a new face and talk. The final sample had nine patients. All participants were above the age of 74. The selection of participants was inspired by the reviews published in the initial phase of this study (Toppenberg, & Uhrenfeldt, 2012; Larsen, & Uhrenfeldt, 2013). They were evaluated by the head nurse as mentally present. The observations began when the patients awoke in the morning before they got up, and for as long as possible throughout the day, ranging from 7:45 a.m. to 8 p.m.

### Data analysis

To bring forward the participants’ experiences is also to ask for their thinking and their “reflexivity by interiority” (Gadamer, 2004, p. 56). The data by which the interpretation starts were not data of experiments and measurements, but historical knowledge served by the participants in unities of meaning through bodily action and language (Gadamer, 2004). Through the observations meaning, units of experiences were captured (Gadamer, 2004) from drawings of settings; sentences written down as they were spoken out in the room or to someone; or through notes on non-verbal language through change of colours in face, tears, sounds of pain, or holding on to parts of the body where, that is, pains were present.

The first step of analysis was to produce a brief overview of the sample by organizing data (Emerson, Fretz, & Shaw, 1995). Second, all field notes from observations were transcribed into coherent text pieces representing each participant. Third, these were read individually and together to identify repeating and deviating patterns to develop sensitizing concepts that, through the process of analysis, became more definitive concepts (Gadamer, 2004) about care challenges of old surgical patients during admission to hospital. Fourth, the theme was developed, scrutinized, and refined through movements between the text pieces from each informant and the texts as a whole, developing three sensitizing subthemes (Hammersley & Atkinson, 2007). Fifth, the themes were illustrated through individual case stories illustrating the situations these themes included, and providing the basis for the analytical generalization in the findings (Miles & Huberman, 1994).

## Findings

We found that different interactions and care challenges influenced the well-being of old hospitalized patients in fast-track hospital treatment. All patients were either being treated for cancer coli (*n=*7) or in the process of being diagnosed for cancer coli (*n=*2). Five were married (men=3, women=2), three were widows/widowers (women=2, men=1), and one woman was unmarried. We identified three main care challenges from the patients’ perspectives: “well-being as a matter of different perspectives,” *“*vulnerability in contrast to well-being,” and *“*staff mix influences on the care encounter.” The challenges are exemplified below to provide a more coherent insight into the understanding and reasoning of older patients.

### Well-being as a matter of different perspectives

The following story illustrates different perspectives of care and interaction and how this relates to Anna. To understand Anna's own perspective of a good life in relation to her recent experience, she introduced herself as a widow living on her own in a small townhouse. Until now, she had been socially very active with friends, children, and grandchildren. Anna continued her story: “Two years ago, a tumour (cancer coli) was reported from the operating surgeon as successfully removed from my colon without any need for further treatment. However, shortly after the operation, I developed an infection in the area where they operated me. I was discharged as planned but I needed to stay at home for the daily routine of cleaning up the infection by the home care nurses. Since then, I have been regularly in and out of the hospital.”

Anna was recently discharged without fever which staff interpreted as a downsized inflammation in the abscess, but the very same evening, her temperature rose and 2 days later, she was readmitted with an active mass in the cavity, due to a new intestinal outlet from the surgical wound. According to Anna, the consultant told her by readmission that: “I was one of 100 that were not sufficiently “stitched” together. Well, normally, my skin heals up very well, so I wonder if this statement can be true or something else could be the matter ….”

During the last 3 months, Anna had spent 8 weeks in hospital within different admissions. During this particular hospital admission, and likewise at home before the admission, the cavity was cleaned twice a day by a nurse or a physician to check the drainage function, the amount excreted and its colour and odour. Therefore, for a long while, her daily activities were planned around this cleaning schedule. To describe how seriously the infection had affected her social life, Anna said with a sad smile and teary eyes: “I've cancelled my trip to the North Pole in June; my family Easter trip was also cancelled as well as the confirmations of two of my grandchildren this spring; and the 70th birthday of a friend, which I had looked forward to participating in for months.”

Anna usually travelled regularly around Europe and previously participated in many kinds of celebrations and events. She was worried about disappointing friends and family members.

Anna further talked about her interactions with staff during the last 24 h. The day prior to the interview, she was under anaesthesia twice: first, due to a gastroscopy, and second, on the same day, when the abscess was inspected and revised. As we spoke, she awaited the consultant's round to learn more about the results from the procedures. “Yesterday, there was just one (doctor) who came by for me after the examination, and told me that there is no doubt that the abscess is related to the gut—but now we will see what they are going to do.”

Anna anticipated that the consultants would be able to predict the development and her outcome of the recent treatment. Their interaction was in this way an intersubjective movement that would positively affect Anna's experience of well-being if the consultant could give her hope of renewed bodily comfort and the possibility of more mobility based on her own preferences.

Nurses, according to Anna's account, were purely focussed on the infection and her physical strength in relation to the healing of the wound. They sought to increase this by pushing her to be physically active, take the prescribed medication, and eat and drink a specific amount and quality per day. She talked about how this made her feel like an object that the staff pushed forward following their own professional aims rather than accepting her present feelings of being unable to increase her mobility. She explained that her bodily discomfort and pain were related to the daily double cleaning process. In addition, it was painful that in-between, there was flatulence in the abscess, which added to the internal pressure in the infected cavity. This pressure was increased by walking and sitting and leaning forward. She experienced that the nursing staff continuously pushed her with encouraging statements as: “Walk another round or two before lunch, maybe together with the researcher …” (field observation).

Anna felt the staff ignored her pain during movements and her sense of suffering that activated her vulnerability. Field observations in the four-bed room where Anna was staying revealed that staff evaluated patients to be in general progression when they were able to sit in a chair during a meal. However, patients like Anna, with recent surgical wounds, experienced this as unpleasant or even explained that it is “impossible to sit on a wound.” As interpreted from the dialogue between nurse assistants and patients in the room and registered nurses (RNs) walking in and out of the room, the questions and expected answers by the staff all concerned how much a patient was able to walk around the ward during the day, etc., reflecting a particular professional demand for physical progress.

Another supportive practice was observed among fellow patients as a way to cope with the demands for physical activity from staff. One of Anna's fellow patients said: “Anna in the third bed (of four) was so exhausted that she needed us to walk her down to the dining room, she was also embarrassed to show her drainage bag from the fistula, we helped her to fix a piece of cloth around it, then she felt fine to go there with us.”

Fellow patients seemed to keep a supportive eye on each other; this way of shared feelings of pain and mutual suggestions of possible comforts seemed to be a result of being in a four-bed room. Based on field observations, it became clear that humour and mutual laughter regarding their present situation relaxed the atmosphere for a while, for example, recognizing the struggle of a fellow patient to get out of bed and joking about it.

### Vulnerability in contrast to well-being

Harry's story reflects the theme of vulnerability during hospital admission. Harry was a 78-year-old widower without relatives or children. He used to be a farmer and now lived on his own on the farm. A neighbour did his grocery shopping, and picked him up every Wednesday for dinner at their house. Basic daily needs (bathroom visits, move from bed to chair, from chair to bed, clean sheets, dinner service) were supported twice a day by the community service. This statement summed up his social life: “At home, the home care people come in at around 9–10 a.m. They serve me some breakfast and help me from my bed into my armchair by the TV—in that chair I sleep the rest of the day away. They return to get me back into bed by 7 or 8 p.m.”

At home, Harry wore a pager around his neck, which by the push of a button gave direct access to home care. Harry had other house calls: home care service cleaned parts of his house every fortnight; and once a week, a nurse delivered his medication divided into weekday doses in a box and further divided into morning, noon, afternoon, and evening doses.

Harry was lying skinny and pale in his bed when the researcher met him, and as we talked, he kept dozing off. When asked if he had the energy to answer questions and be followed around all day, he answered: “Yes, definitely, I want to contribute to your research.”

Then he closed his eyes again. After some small talk concerning why he had been admitted to hospital, we talked about how he felt about the upcoming discharge and he responded: “The loneliness at home is hard, nobody to talk to; that is the worst part of it.”

Older patients treated surgically for cancer experience a general setback in energy. Some of this may be helped by protein-enriched drinks, food, and pain killers. For others like Harry, the weight loss is too hard to regain, influencing their mood. Their aim and purpose of life as well as the relations and expectations towards the staff are reduced to the patients receiving what the staff offers and not requesting anything in particular (19, 25). When asked, Harry said he never read the daily menu card in the ward. Upon realizing that his favourite dish (apple porridge) was displayed on the menu as an option, he decided to get out of bed and eat in the chair. Harry's profound suffering was the care challenge here, not limited only to his bodily discomfort and pain, but also progressed immobility leaving a number of activities that he now was unable to do on his own.

Such suffering and discomfort may be difficult to verbalize by patients as it requires words about personal existential experience, but were accessible here through the prolonged observation. This may challenge the care interaction with patients like Harry, as staff is mainly relying on what the patient is able to verbalize.

### Staff mix influences the encounter

Field observations revealed that the nursing staff mix influenced the encounter with patients. The members of staff were the ones to set the criteria and evaluations that discharge was based on, and the arguments for discharge were characterized by a different reasoning than the needs experienced by patients. Patients concluded this as a requirement to progress hospital economy. Anna recalled: “Last time I was here, they said that they arranged for me to be discharged with home care because they could not afford to have me admitted here any longer. Well, I would rather stay here until I was healthy again. They said my strength was good enough to come back home.”

Patient well-being before discharge seemed only to be evaluated by staff. On the days of the field observations with Anna and Laura, the staff members present were a nursing assistant (NA) with 2 years of diploma courses, two RNs, one occupational therapist, a kitchen assistant, and a consultant who made visits to specific patients. The nursing staff shared tasks such as serving breakfast; encouraging patients to get out of bed early; and supporting them during visits to the bathroom, walking down to the dining room, moving around in the hospital during the day, and preventing them from staying in bed or in a chair. During the day, whenever the RNs visited the individual patients, their agenda was to deliver a message, to prepare discharge, or to inform the patients about changes in their treatment (field observations). All staff routinely requested patients, in what patients interpreted as a firm voice, to move from bed to chair, from chair to floor, from floor to bathroom, and from chair in bedroom to living room rather than staying in bed. Their approach to the daily patient routines, however, was very different. Some staff debated with patients, whereas others just told patients that is was time to get out of bed (field observations).

Field observations with patients revealed that they did not find the staff able to understand how they as patients experienced their present situation. The staff was described as uncaring by the patients. After an incident, where a nursing student asked a patient “to get up from the chair and walk around” (field note), the patient (Laura) expressed loudly how she felt nauseous and in pain and was met by the nursing student only with a mumble as reply. It seemed difficult for the nursing student to interpret this situation independently without guidance from senior staff. She kept standing in front of the patient and then after a while left the room without fulfilling her task; as she left the patient looked at her fellow patients with eyebrows raised and no words, indicating that this new nurse student was just another burden to be endured.

Carried by the argument that she needed the exercise, a NA asked Laura in the morning to move from bed and into a chair during breakfast (instead of having breakfast in bed), and then to move on to the bathroom and living room. Laura expressed that normally she lived an active life with contact to her daughters living in the same city as herself. Usually Laura, as she saw herself, was a happy person. However, at our first meeting, she was close to crying. Her mouth trembled and her eyes were wet, she was still in bed and had not yet eaten breakfast. She looked me straight in the eyes and asked if she could still cancel our agreement if she felt too tired, which I confirmed. Our conversation gradually moved from small talk about the reason to do this study and why her contribution could inform this aim into what seemed to be her primary message. With a firm tone she argued: “There are two kinds of nurses, those who are caring and those who show off their power.” She referred to nurses in general (RN, NA, nursing student) who did not accept her need to relax in bed and to keep the belly area around her wound in peace to prevent nausea. Her argument for staying in bed was that after the surgical removal of the anus and the establishing of her colostomy, the pain increased whenever she tried to sit down, and provoked nausea and dizziness. Nurses did not accept this as a valid reason for her to stay in bed. Laura did not have the strength to keep arguing with them, she said: “It takes too much effort to argue with such an uncaring person, so much effort that it feels insurmountable at the moment when it occurs.”

For Laura, movements were painful because they pushed on her wounds and provoked the pain inflicted by her colostomy, which was still a bit swollen. This led to nausea and a feeling of an overwhelming weakness and sadness. A feeling of being in an uncaring atmosphere and the lack of personal progress made Laura feel more tired. However, from a clinical stance (regulations and guidelines), the staff's task was to document the patients’ progress through their treatment, their progress in physical mobility, and patients’ specific insights regarding how to gain additional strength in the time after discharge. Therefore, a lack of positive interaction between staff and patients may result in a managerial reaction due to delayed discharge, and a negative influence on the department's waiting lists and economy associated with the number of patients treated.

## Discussion

This study explored how the well-being of old hospitalized patients is affected by, and is dependent on, the interaction with staff during a hospital admission for surgical treatment of colon cancer.

From the patients’ perspective, their well-being during and after hospitalization was highly dependent on a range of interactions they had with the healthcare staff. All these interactions reflected a clash of perspectives between staff and patients. The individual nurses’ discernment and encouragement was experienced by patients as personal manifestations of power, which seemed to strongly affect their sense of well-being. Such divergence between the acts and encouragements of the staff to the perspectives of the patients on the tasks (e.g., mobilization) they were required to perform reflects the ambivalence of what Mol (2008) has termed the “logic of care”. As staff and patients interact on a shared task related to the care of the patient, it is clear from our findings that their motivations are quite different. Where the nurse is motivated by her professional knowledge of, for example, the need for physical movement in healing after surgery, the patient is motivated by a personal concern of pain and strain to minimize discomfort.

Patients, as reflected by the case of Anna, evaluated well-being by their own sense of healing and progression and were suspicious towards clinical explanations of why healing did not progress as expected, sensing that this might only be an excuse for not providing optimal treatment and not the truth. Such sceptical encounters shaped the future orientation to well-being in patients, confirming their own knowledge of their bodies, and in the context of such experience makes patients openly wonder if the judgement of the healthcare staff concerning progress or lack of health progress can be trusted. As exemplified by the three patient cases described in this study (Anna, Harry, and Laura) to illustrate the care interactions with old cancer patients in fast-track surgery, well-being was not experienced as central in the interaction between patient and staff. The key focus of staff was to encourage physical mobility and discharge.

Galvin and Todres (2011) write of dwelling as a contrast to mobility; in an ideal world, they both exist and are intertwined by the patients’ choices. In this study, dwelling was not observed; however, it was addressed when patients in different ways addressed their need for rest in the bed, the chair, or at home with their spouse. Among fellow patients, the vulnerability was recognized and supported. It is thought-provoking that it takes fellow patients to acknowledge a patient's embarrassment of wearing a visible drainage bag to the dining room. Furthermore, the mobility required to reach the dining room for a nauseous patient may result in appetite loss, leading to a reduction in the intake of protein-enriched food.

Our findings revealed that the nursing staff mix at the ward affected the experience of care in different ways. However, there are no national policies concerning the best nursing staff mix in many European countries (Attree et al., 2011). While the RNs came to the patients’ room for short messages or visits, the nursing students or nurse assistants seemed to be in charge of the patients’ progress towards discharge, evaluated as their general mobility and the distances they were able to cover after the surgical intervention. The tone of voice used by staff members was experienced as very revealing by patients as to the intentions of staff as caring or uncaring. A student or assistant using a firm voice rather than a caring voice may have expressed a lack of professional competence, which could explain why they seemed to act as novices following a set routine, instead of acting in a more proficient situational interaction with the patient (Benner, Tanner, & Chesla, 1996, 2006).

Anna's subjective experience of her bodily discomfort and pain during advised physical mobilization not being recognized by the healthcare staff, when asking her to move around, led to an immense experience of suffering. To the observer, it seemed thoughtless that the staff followed routines for mobilization of surgical patients when these patients all suffered additional problems following their cancer, which possibly delayed the healing of wounds. Arendt, in her description of how human beings testify the presence of each other, clarifies that “Absence of thought is indeed a powerful factor in human affairs, statistically speaking the most powerful, not just in the conduct of the many but in the conduct of all” (Arendt, 1971, 1998, p. 71). Following this line of thought, there is a difference between truth and meaning. Truth being based on knowledge, and meaning being based on thinking (Gadamer, 2004, p. 62), there may be a risk that nursing practice only built on scientific facts, as the need for mobilization to ensure a positive outcome of fast-track surgery omits a caring practice that seeks to recognize what gives meaning for patients (Arendt, 1971, 1998). A thoughtless presence (Schanz, 2007) may turn out to be experienced or observed as cruel, or use of unnecessary power, if the staff show no interest in the patients’ thinking and follow routines or guidelines bypassing the individual patients’ reactions, needs, age, etc.

Galvin and Todres (2013) describe it as suffering when a person feels trapped, left with no horizon, and maybe even a kind of spatial imprisonment. The accounts of the older surgical patients to some extent reflected such suffering and lacked perspective for healing. A caring, attentive, nursing practice may have identified such suffering and started counteracting it through interactions based on recognition.

### Limitations and strengths

The first author was required by the hospital patient safety regulations to wear an average nurse uniform during the field study. However, this did, to some extent, position the researcher as a nurse in the interactions and shaped the encounter between staff, patients, and researcher during data collection in certain ways, for example, by staff members counting on the researcher to fulfil certain care duties.

Recognizing this imminent need, the researcher did engage in the care interaction at certain moments, such as walking the patients up and down the ward, serving meals, helping a frustrated patient to the toilet, and picking him up again when nobody seemed to notice his bell. These were acts of duty made possible by the uniform but may also have occurred due to the uniform. Though such interactions affected the researcher's position to some extent and may have limited the study in some ways, they also opened potential insights into the patients’ experience of care that would not have been possible otherwise. Our engagement of the theoretical framework of well-being and caring in the analytical work with the three specific cases allows for an analytical generalization, as is common to qualitative research (Halkier, 2011).

## Conclusion

The care challenges in the interaction between old hospitalized cancer patients and nursing staff are dependent on how the staff interacted with patients when they followed routines and guidelines.

The central focus to staff was to encourage physical mobility and discharge. However, their approach differed according to their professional experience. Patients experienced being on their own regarding pain, nausea, or general vulnerability; well-being did not seem to be a key element of the interaction between patient and staff. Fellow patients recognized vulnerability and supported each other.
